# Setting the Tempo in Development: An Investigation of the Zebrafish Somite Clock Mechanism

**DOI:** 10.1371/journal.pbio.0050150

**Published:** 2007-05-29

**Authors:** François Giudicelli, Ertuğrul M Özbudak, Gavin J Wright, Julian Lewis

**Affiliations:** Vertebrate Development Laboratory, Cancer Research UK London Research Institute, London, United Kingdom; Cambridge University, United Kingdom

## Abstract

The somites of the vertebrate embryo are clocked out sequentially from the presomitic mesoderm (PSM) at the tail end of the embryo. Formation of each somite corresponds to one cycle of oscillation of the somite segmentation clock—a system of genes whose expression switches on and off periodically in the cells of the PSM. We have previously proposed a simple mathematical model explaining how the oscillations, in zebrafish at least, may be generated by a delayed negative feedback loop in which the products of two Notch target genes, *her1* and *her7,* directly inhibit their own transcription, as well as that of the gene for the Notch ligand DeltaC; Notch signalling via DeltaC keeps the oscillations of neighbouring cells in synchrony. Here we subject the model to quantitative tests. We show how to read temporal information from the spatial pattern of stripes of gene expression in the anterior PSM and in this way obtain values for the biosynthetic delays and molecular lifetimes on which the model critically depends. Using transgenic lines of zebrafish expressing *her1* or *her7* under heat-shock control, we confirm the regulatory relationships postulated by the model. From the timing of somite segmentation disturbances following a pulse of *her7* misexpression, we deduce that although *her7* continues to oscillate in the anterior half of the PSM, it governs the future somite segmentation behaviour of the cells only while they are in the posterior half. In general, the findings strongly support the mathematical model of how the somite clock works, but they do not exclude the possibility that other oscillator mechanisms may operate upstream from the *her7*/*her1* oscillator or in parallel with it.

## Author Summary

Somites—the embryonic segments of the vertebrate body—are formed sequentially, with a spacing determined by a gene expression oscillator, the segmentation clock, operating in the cells at the tail end of the embryo. This system provides a rare opportunity to analyse how the timing of at least one set of developmental events is controlled. We previously proposed a mathematical model, showing how the oscillations could be generated by a delayed negative feedback loop, in which the products of two genes, *her1* and *her7,* act as inhibitors of their own expression, and how Notch signalling between adjacent cells keeps their individual oscillations synchronised. Here we test and find support for this model in two ways. First, we show how to use the spatial pattern of gene expression to measure some of the temporal delays and molecular lifetimes that are critical for the occurrence of synchronised oscillations. Second, we use transgenic fish in which expression of *her1* or *her7* can be switched on at will by heat shock to probe the dynamics of the system and to analyse the logic of the control circuitry.

## Abstract 3

The vertebrate somites are laid out in a regular pattern determined by the segmentation clock. Here the authors verify a mathematical model of the clock model and show that it quantitatively fits experimental details.

## Introduction

How do genes set the tempo of embryonic development? This basic question is still largely unanswered. We lack quantitative information about the dynamics of gene expression in the embryo, and in most cases, we do not even know which genes govern timing, let alone how they do so. In this paper, we focus on one particular process, zebrafish somite formation, in which a better understanding may be attainable: previous work has identified specific genes as critical for the control of timing, and a detailed mathematical model has been proposed to explain how they could act as a timer. Our goal is to test this model through measurements of the dynamics of the real system.

**Figure 1:**
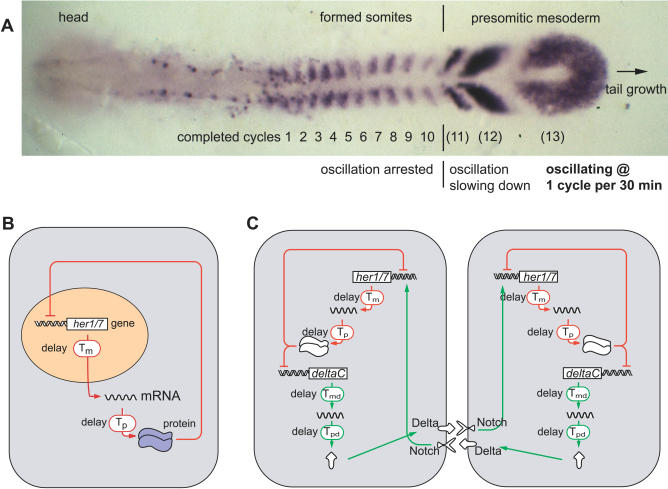
Mapping of Temporal Oscillations into a Spatial Wave Pattern during Somite Segmentation, and a Model of the Oscillator Mechanism (A) The expression pattern of the oscillating gene *deltaC* in a zebrafish embryo at the ten-somite stage. (B) In each cell of the presomitic mesoderm, it is proposed that a *her1* or *her7* autoinhibition negative feedback loop generates oscillations. (C) Communication via the Delta-Notch pathway is proposed to keep oscillations in adjacent cells synchronized. The oscillations depend critically on the delays (*T*_m_, *T*_p_, *T*_md_, and *T*_pd_) in the feedback loops. (A–C) are slightly modified from [25].

The somites—the future segments of the vertebrate body axis—are laid down sequentially by a mechanism involving oscillating gene expression in the cells of the presomitic mesoderm (PSM) (see Figure 1A). Here, at the tail end of the embryo, the transcripts of certain genes undergo regular coordinated cycles of production and degradation [1–9]. This gene expression oscillator is called the somite segmentation clock, and in each of its cycles, one additional somite is formed. The cycling is rapid: in the zebrafish at 28 °C, the period is 30 min. A gradient of FGF8, with its high point in the tail bud where the *Fgf8* gene is transcribed, and acting in opposition to retinoic acid released by anterior tissues [10–14], appears to define the extent of the PSM and thus the region within which cycling continues. As the cells in the PSM proliferate and the tail bud grows caudally, the maturation “wavefront” delimiting the region of high FGF8 concentration moves caudally also, causing the cells in the anterior region of the PSM to slow down and finally cease their oscillations as they emerge from the PSM and begin differentiation. The gradual slowing of the oscillation in the anterior part of the PSM is manifest there in a spatial pattern of travelling stripes of gene expression. Cells that are in different phases of the oscillation cycle as the maturation wavefront sweeps over them become arrested in different states, corresponding to expression of different genes and apparently defining which portion of a somite they will form. The succession of cells passing through the anterior part of the PSM to begin their differentiation as somites can thus be likened to magnetic tape passing the recording head in a tape recorder: the periodic somite pattern represents a spatial record of the temporal oscillation in the posterior PSM. It is this spatiotemporal map that makes the system particularly attractive for investigation of developmental timing.

What, then, is the mechanism of the intracellular oscillation, and how are the oscillations of adjacent cells coordinated? All of the mutations known to specifically disrupt the PSM oscillations in zebrafish lie in components of the Notch signalling pathway [15–18] (although in mammals and birds, there is evidence that the Wnt/beta-catenin and other pathways are also involved [1,3,4,9]). Previous work in the zebrafish has shown that a closely related pair of Notch target genes, *her1* and *her7,* are strong candidates for a central role, along with the Notch ligand gene *deltaC*. *her7, deltaC,* and perhaps *her1* are required for coordinated oscillation of other markers and for regular somite segmentation, and all three show oscillating expression in the PSM in synchrony with one another [16,19]. *her1* and *her7* both belong to the Hairy/E(spl) family of putative transcriptional repressor genes, several of which are regulated by, and regulators of, Notch signalling [16,19–22]. Notch signalling thus provides a communication mechanism to synchronize the *her1*/*her7* oscillations of adjacent cells [6,23–25]. Moreover, *her1* and *her7* are thought to negatively regulate their own expression [19]. This suggests that a negative feedback loop based on direct autorepression of *her1* and/or *her7* could be the fundamental pacemaker mechanism of the somitogenesis oscillator (Figure 1B). But is this really the case, and if so, how is the period of oscillation specified? To answer these questions, intuition is not enough: one has to work out the underlying mathematics and analyse the system quantitatively.

We have shown by mathematical modelling [25,26] that the possibility of oscillation in such a simple negative feedback system depends critically on the delays involved in transcription and translation—that is, the time *T*_m_ that elapses from initiation of a transcript to its emergence into the cytosol as a mature mRNA, and the corresponding time *T*_p_ from initiation of translation to delivery of the functional protein molecule to its site of action. When these delays are taken into account, theory predicts that autoinhibition of *her1* or *her7* (or of both together) will give rise to robust oscillations, provided certain conditions are satisfied: in particular, the lifetimes of the Her1/Her7 protein and mRNA molecules must both be short compared with the sum of delays *T*_m_ + *T*_p_. The predicted period of oscillation *T,* if oscillation occurs, is then given by the simple formula:

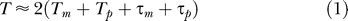
where *τ*_m_ and *τ*_p_ are the lifetimes of the mRNA and protein, respectively. This analysis explains the pattern of somite defects in mice in which the lifetime of the Her1/Her7 homolog Hes7 has been artificially lengthened [27].

The theory predicts, furthermore, that the oscillations in adjacent cells will be kept synchronized through communication via the Delta-Notch pathway (Figure 1C), but again only if certain conditions are met. Experimental evidence indicates that the Her protein regulates expression of the *deltaC* gene in parallel with that of the *her* gene, and that activation of Notch by DeltaC contributes to regulation of *her* gene expression [15,19]. In the corresponding mathematical model, synchronization then requires a relatively large delay, of the order of one oscillator cycle, in the Delta-Notch signalling pathway—a delay that includes the translational plus transcriptional delay for DeltaC. This delay is indeed expected to be long because of the time needed for delivery of DeltaC to the plasma membrane via the secretory pathway.

Taken all together, the experimental evidence suggests a simple mathematical model of how specific known genes may act as pacemakers and synchronizers of the somite segmentation clock. This model oscillator system will only work, and will only fit the observed oscillation period, if certain parameters have appropriate values. Previously, we inserted rough estimates, based on rather scanty data available from other systems, and argued that the theory was quantitatively, as well as qualitatively, plausible. Nevertheless, its validity rests on several unproven conjectures. These concern both the detailed logic of the regulatory interactions between *her1, her7,* and *deltaC,* and the actual values of the biosynthetic delays, molecular lifetimes, and other parameters for the cells in the zebrafish PSM.

We have therefore set out to check these conjectures experimentally, through artificial manipulation of the components and through measurement. To probe the dynamics of such rapid transcriptional oscillations, one needs a rapid way to switch expression of the individual genes on or off at will. For this purpose, we have generated transgenic lines of zebrafish containing an inducible cassette of either *her1* or *her7* under the control of a heat-shock–responsive promoter. We have used these and other methods to examine the following questions:

Do the oscillating genes *her1, her7,* and *deltaC* show transcriptional and translational delays of the magnitudes postulated by the theory? Do the gene products—the mRNAs and the proteins—have the postulated short half-lives? What is the regulatory circuitry linking the oscillating genes? Do the oscillations recover after resetting by overexpression of *her1* or *her7,* and if so, how rapidly? Finally, when *her1* or *her7* is transiently overexpressed, what is the time course of resulting somite segmentation defects? In other words, how does expression of these oscillating genes affect somite segmentation, and at what point in the history of a given somite do they act?

## Results

### The Temporal Sequence of Events in the Clock Cycle Is Mapped Out Spatially in the PSM

The spatial wave pattern seen in a fixed specimen can be used to derive information about the temporal oscillations that generated that pattern in the living tissue. For this purpose, we need first of all to understand quantitatively how the spatial and temporal patterns are related.

**Figure 2:**
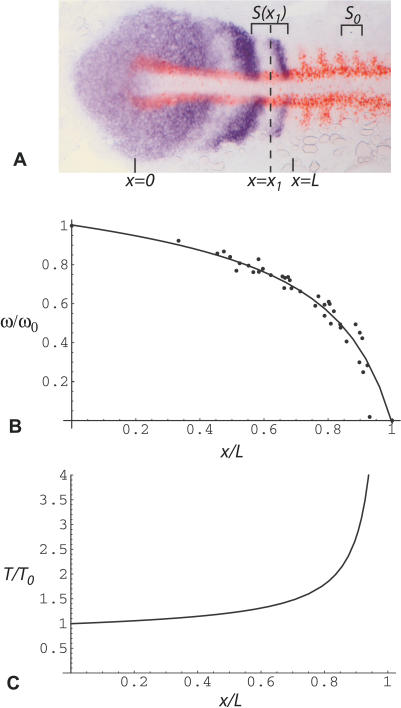
Estimation of Clock Rate *ω*(*x*) and Period *T*(*x*) as a Function of Distance *x* along the PSM (A) Flat mount of a seven-somite embryo stained by ISH for *her1* (purple, NBT/BCIP detection chemistry) to show the oscillation pattern and for *myoD* (red, Fast Red detection chemistry) as a marker of formed somites. Spatial wavelength as a function of position, *S*(*x*), is measured as the interval between one *her1* peak and the next, or one trough and the next, where *x* is the midpoint of that interval. *S*_0_ is the width of one formed somite. Position *x* = 0 corresponds to the tail end of the notochord, and position *x* = *L* corresponds to the anterior end of the PSM. (B and C) Clock frequency *ω*(*x*) and period *T*(*x*) as a function of distance *x* along the PSM. Each data point corresponds to one measurement of spatial wavelength *S*(*x*). The graph represents data from ten different, randomly chosen specimens of the type shown in (A) fixed at the 7–15-somite stages. The line (a hyperbola) is a least-squares fit to the data. To combine data from different specimens, distances are scaled relative to the length of the PSM. Clock rate and period are calculated for each data point according to the Equation 2 shown in the text. We use a simple linear approximation for *u*(*x*), the speed of forward movement of the cells along the anteroposterior axis relative to the tail end of the notochord: by definition, at *x* = 0, *u*(*x*) = 0, and at *x/L* = 1, *u*(*x*) = 1 (in somite lengths per somite cycle), so we assume *u*(*x*) = *x/L* at intermediate values of *x*.

At the posterior end of the PSM, oscillations of gene expression occur at a frequency *ω*_0_ of one cycle per 30 min, corresponding to the time taken to form one additional somite. At the anterior end of the PSM, oscillation is arrested. In between, the oscillation slows down gradually. This means that anterior PSM cells are retarded in oscillator phase relative to posterior PSM cells; the retardation is greater the further anterior the cells lie. As a result, one sees the different phases of the oscillator cycle mapped out in space along the length of the PSM, where they are manifest as stripes of expression of the oscillating genes [15,23,28,29]; the number of stripes reflects the number of cycles by which cells at the anterior end of the PSM are delayed relative to the cells at the posterior end. We can exploit this spatial graph of the oscillation cycle to make measurements of the timing of oscillator events. To do so, however, we need a precise statement of the relationship between position and oscillator phase along the length of the PSM. This depends on the rate at which the clock runs in the cells in the different positions, and on the way in which the cells move as the tail bud extends and additional somites form. For cells close to the midline, whose movement is oriented along the anteroposterior axis, the formula is as follows (see Materials and Methods for a derivation):

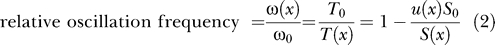
where *ω*(*x*) is the frequency of the oscillation (in cycles per unit time) in the cells at the given position *x* along the axis, *T*(*x*) = 1/*ω*(*x*) is their oscillation period, *T*_0_ = 1/*ω*_0_ is the somite cycle time (the period of the fundamental somite segmentation clock, i.e., 30 min for the zebrafish at 28 °C), *u*(*x*) is the velocity of the cells relative to the tail bud measured in somite lengths per somite cycle, *S*(*x*) is the local spatial wavelength (the distance from one peak to the next or one trough to the next) measured along the trajectory of the cells, and *S*_0_ is the length of a formed somite. As shown in Figure 2, we can use this formula to plot a graph of the oscillation period *T*(*x*) as a function of distance along the anteroposterior axis. From the graph, we see, for example, that halfway along the PSM, *T*(*x*) ~ 1.2 *T*_0_, while at a point anterior to this, three quarters of the way along the PSM, *T*(*x*) ~ 1.6 *T*_0_.

If two events of the oscillation cycle appear separated by a distance *δx* in a snapshot of the PSM, we can deduce that in the history of a given cell, as the cell moves through the PSM, the events are separated in phase by a fraction *δx*/*S*(*x*) of the local cycle, i.e., by a time interval

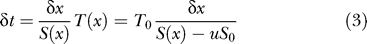



Similarly, if the concentration *C* of some molecule in the cells changes as a function of the cells' phase in the oscillation cycle, we can deduce its rate of change with time within a given cell (i.e., its material derivative, D*C*/D*t*) from the rate of change from cell to cell with respect to position at a given instant:

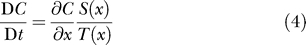



We can use these relationships to deduce the timing and rate of processes in the intracellular cycle from snapshots of the spatial pattern of cells in different states in the PSM.

### *her1, her7,* and *deltaC* Show Transcriptional Delays as Predicted

**Figure 3:**
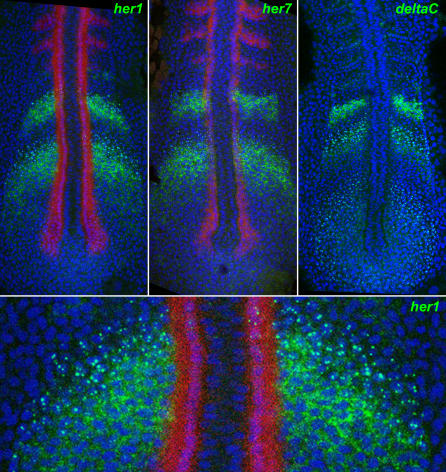
Nascent and Mature Transcripts Visualized by In Situ Hybridisation Fluorescent staining by ISH for *her1* (top left)*, her7* (top middle)*,* and *deltaC* (top right)*,* using tyramide chemistry (green), reveals nuclear dots corresponding to nascent transcripts and cytoplasmic signal corresponding to mature mRNA. The bottom panel is a magnified detail of the top left panel. The images are confocal optical sections of flat-mounted specimens, counterstained for DNA with TOPRO3 (blue false colour). Red staining shows *myoD* expression by dual ISH.

We used fluorescent in situ hybridisation (FISH) as described in Materials and Methods to analyse the expression of the cyclically expressed genes *her1, her7,* and *deltaC* (Figure 3). For each of these genes, two types of in situ hybridisation (ISH) signals were visible. Some cells showed diffuse labelling in their cytoplasm, corresponding to cytoplasmic mRNA molecules. Other cells showed intense dots in the nucleus, corresponding to transcripts in the course of synthesis; often such dots occurred as pairs, corresponding to the two gene copies present in G1 phase of the cell division cycle, but in some nuclei, only one dot was seen (either because other dots, though present, were not included in the optical section, or because of the stochastic nature of transcriptional control), and in some nuclei (presumably in G2 phase), there were three or four dots.

**Figure 4:**
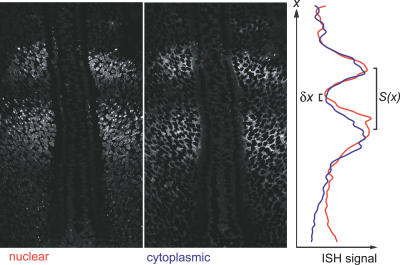
Estimation of Transcriptional Delay from In Situ Hybridisation Pattern The spatial offset between the anterior margin of the band of nuclear dots and the anterior margin of the band of cytoplasmic signal in specimens stained as in Figure 3 gives a measure of the transcriptional delay. To measure this offset, we used Photoshop to generate from the image of each specimen a pair of pictures, one (left-hand panel) showing only the ISH signal that was nuclear (i.e., co-localized with DNA staining), the other (middle panel) showing only the signal that was cytoplasmic (i.e., co-localized with an absence of DNA staining); note, however, that because of the non-zero thickness of the optical section, the “nuclear” signal includes a sizeable contribution from cytoplasmic mRNA where the latter is plentiful. For each of these two images, we computed the smoothed mean intensity of staining as a function of distance *x* along the anteroposterior axis (see Materials and Methods), and plotted the results together on the same graph (right-hand panel). The delay from the beginning of the rise in nuclear signal to the beginning of the rise in cytoplasmic signal corresponds to the spatial offset *δx* between the minima of the red and blue curves. We converted this offset to a time interval using Equation 3 and the local values of *x, L, S*(*x*), and *S_0_* measured from the same specimen.

The bands of cells with nuclear dots lay slightly anterior to the bands of cells with cytoplasmic staining, and from this spatial shift, we can estimate the delay from the time when nascent transcripts first become detectable in the nucleus to the time when the mature transcripts first become detectable in the cytoplasm (Figure 4). This delay represents a lower bound to the transcriptional delay *T*_m_ as defined in the mathematical model: it leaves out of account the time *T*_init_ that must elapse from disappearance of the inhibitory signal (free Her1/7 protein in the nucleus) to the appearance of detectable nascent transcripts. *T*_init_ includes time required for bound inhibitory protein to dissociate from the DNA and time for the RNA polymerase to generate sufficient transcript to be recognised by the ISH probe. Adding *T*_init_ (whose value we do not know) to the delays manifest in the ISH pattern, we arrive at the following estimates for the transcriptional delays: for *her1, T*_mher1_ = 3.8 ± 1.0 min + *T*_init-her1_ (mean ± standard error of the mean [s.e.m.], *n* = 10); for *her7, T*_mher7_ = 3.7 ± 1.4 min + *T*_init-her7_ (mean ± s.e.m., *n* = 7 ); and for *deltaC, T*_mdeltaC_ = 8.4 ± 1.2 min + *T*_init-deltaC_ (mean ± s.e.m., *n* = 7).

The numbers are probably more trustworthy for *her1* and *deltaC* than for *her7,* for which the in situ staining intensity was rather weak. These values can be compared with those, based on completely different and much less-direct evidence, that we used previously in computing the behaviour of our mathematical model: there we assumed *T*_mher1_ = 12 min, *T*_mher7_ = 7.1 min, and *T*_mdeltaC_ = 16 min (see [25], Figures 3 and 4). The new values would thus roughly agree with the old if *T*_init_ were of the order of 3 to 8 min.

### The Spatial Pattern of Transcript Concentrations in the PSM Provides Information on Transcript Lifetimes

In a snapshot of the PSM, the steepness of the decline in the number of transcripts per cell as a function of distance going caudally from a peak of the transcript distribution gives a measure of the rate at which transcript concentrations decrease with time in a typical cell as it progresses past the peak of its oscillation cycle. The rate of transcript degradation in a cell must be at least as fast as the observed net rate of decrease in the number of transcripts it contains, and will be faster than this if new transcripts are being produced at the same time. Thus, by analysing the spatial pattern and using Equation 4, we can derive an upper bound to the transcript lifetime, *τ*_m_. The relationship is as follows:

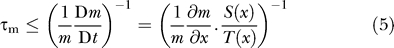
where *m*(*x,t*) is the concentration of transcripts in a cell at (*x,t*), and lifetime is defined in such a way that half-life = lifetime × ln2 ~ 0.69 × lifetime.

We can use our fixed ISH specimens to calculate this upper limit if we assume that the intensity of the ISH signal is proportional to the concentration of transcripts present; details of the analysis are given in Materials and Methods. From specimens stained with tyramide chemistry to give a FISH signal (as in Figure 3), we find *τ*_mher1_ ≤ 6.6 ± 0.8 min (mean ± s.e.m., *n* = 12) ; *τ*_mher7_ ≤ 8.1 ± 1.2 min (mean ± s.e.m., *n* = 8); and *τ*_mdeltaC_ ≤ 6.1 ± 0.6 min (mean ± s.e.m., *n* = 5).

Noise, background staining, and possible nonlinearity in the relationship between mRNA concentration and ISH signal all mean that these numbers are only rough estimates. As a partial check, we also made the same measurements from a set of specimens stained for *her1* using NBT/BCIP chemistry (as in Figure 2). These yield *τ*_mher1_ ≤ 5.6 ± 0.5 min (mean ± s.e.m., *n* = 12).

Given that the actual values of the lifetimes are likely to be shorter than the estimated upper bounds, the above measurements are consistent with the value of 4.3 min that we assigned to each of these lifetimes in computing the behaviour of our mathematical model.

### DeltaC Protein Concentrations Oscillate and Are Delayed by Approximately 30 Min Relative to *deltaC* Transcripts

Although cyclic transcription in the PSM has been well documented in zebrafish, frog, chick, and mouse [1–8], data concerning the dynamics of the corresponding protein products are extremely scarce. Only two or three instances of cyclic protein expression during somitogenesis have been described so far: in the chick PSM, Western blots against the Lunatic Fringe protein have been used to demonstrate its cyclic expression [30]; in the mouse PSM, immunohistochemistry with an anti-Hes7 antibody shows a stripy pattern, which has been taken as evidence for oscillations in Hes7 protein levels [31]; and the level of Notch activation has also been shown to oscillate in the mouse [32]. There is no published evidence as to whether levels of Delta protein oscillate; indeed, of the many studies reporting *Delta1* expression during somitogenesis in chick and mouse, only one [33] has described it as oscillatory at the mRNA level. In the zebrafish, *deltaC* clearly shows oscillating transcription, and it is clear that DeltaC function is necessary for oscillator function [6,15,29]; but the question remains open whether oscillations of DeltaC protein levels are required, or indeed occur at all, in the zebrafish PSM. This is an important issue for our theoretical model, which supposes that oscillating levels of DeltaC protein provide the signals that keep neighbouring cells synchronized.

**Figure 5:**
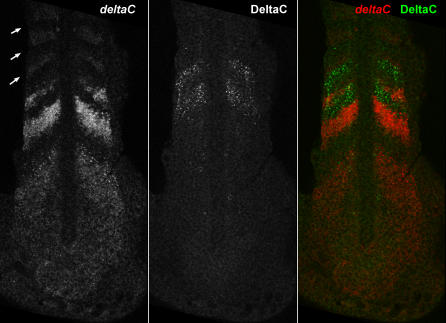
The Pattern of *deltaC* Transcripts Compared with the Pattern of DeltaC Protein in a Doubly Stained Specimen The left-hand panel shows the distribution of *deltaC* mRNA, as revealed by ISH; arrows indicate the three most recently formed somites. The middle panel shows the distribution of DeltaC protein in the same optical section, immunostained with the zdc2 monoclonal antibody. The right-hand panel shows the two patterns superimposed in the doubly stained specimen. The protein pattern is shifted by almost exactly one somite width relative to the mRNA pattern; one somite cycle time earlier, the cells currently displaying peak levels of protein would have been in the locations of the cells currently expressing the peak levels of mRNA and would have been expressing those levels of mRNA themselves. Thus there is a delay of approximately one somite cycle time (30 min) from the accumulation of the mRNA in the cell to the accumulation of the protein translated from it.

To address this question, we used a monoclonal antibody raised against the zebrafish DeltaC protein (zdc2; see Material and Methods). Immunostaining with this antibody revealed stripes of DeltaC protein in the anterior PSM, indicating that DeltaC protein levels do oscillate in this region at least (Figure 5). Levels of DeltaC protein in the posterior PSM were too low to detect confidently, and thus too low for us to see clear evidence of oscillations, but this should not be taken to imply that they were too low to be physiologically important or that they were actually non-oscillatory. Protein degradation in this region is rapid, levels of the mRNA are lower than in the anterior PSM, and we have found in other tissues that the amounts of DeltaC and DeltaD are frequently so small as to be barely detectable by immunofluorescence even where there is clear genetic evidence that they are functionally important ([34] and unpublished data). Presumably, a very small amount of Notch ligand is enough to activate Notch effectively. Although levels of DeltaC protein in the posterior PSM were thus too low for us to demonstrate oscillations clearly, in the anterior PSM there was no such difficulty. In this region, comparison of the DeltaC immunofluorescence pattern with the *deltaC* mRNA pattern allowed us to estimate the DeltaC translational delay (from the time of the beginning of synthesis of a DeltaC molecule to its arrival in intracellular vesicles, presumably via a journey to the cell surface [34–36]). As shown in Figure 5, the pattern of bands of DeltaC protein immunostaining is similar to the pattern of bands of *deltaC* mRNA, but shifted anteriorly by almost exactly one somite width. Because cells take one somite cycle time to move one somite width, this means that the cells that show the peak levels of mRNA (close to the anterior end of the PSM) display peak levels of the protein one somite cycle—i.e., 30 min—later.

In our mathematical model, we found that a DeltaC “translational delay” of the order of 20 min (on top of a transcriptional delay of 16 min) gave good synchronization of adjacent cells. This parameter in the model comprises the delay from initiation of DeltaC translation to delivery of mature DeltaC protein to its site of action at the surface of the signalling cell, plus the delay for signal transduction in the receiving cell (from activation of Notch at the cell surface to its arrival at its site of action in the nucleus). We do not know the size of the latter contribution, though it is likely to be small, because it involves only a protein cleavage event and transport from the cytoplasm into the cell nucleus. There is also some uncertainty as to the timing relationship between availability of DeltaC at the cell surface and presence of DeltaC in endocytic vesicles in the cytoplasm (which is where we chiefly detect the protein with our antibody) (discussed in [34] for DeltaD). With these provisos, the measured delay of 30 min from appearance of peak levels of *deltaC* mRNA to appearance of peak levels of DeltaC protein agrees reasonably well with the delay required in the mathematical model to enforce synchronisation of adjacent cells.

### A Heat-Shock Response Element Drives a Pulse of *her1* and *her7* Overexpression in Transgenic Fish

To test the dynamics of the control system and to check the regulatory interactions between its components, we generated stable fish lines carrying a transgene in which the zebrafish *hsp70* heat-shock promoter was coupled to the cDNA sequence of either *her1* or *her7*. In these fish, we could artificially trigger a pulse of expression of *her1* or *her7* and follow the consequences. The transgene was designed so that the resulting transcript would be similar to the native *her1*/*7* mRNA, including the 3′ untranslated region (UTR), with the 5′ UTR being that of *hsp70*. We also inserted a sequence coding for a haemagglutinin (HA) tag in frame with the N-terminus of the Her protein, so that the transgene product would be easily detectable. By screening fish injected with the appropriate DNA constructs, we identified and isolated stable transgenic lines carrying either *hsp70:HA-her1* or *hsp70:HA-her7*. At least two independent lines were isolated for each construct.

**Figure 6:**
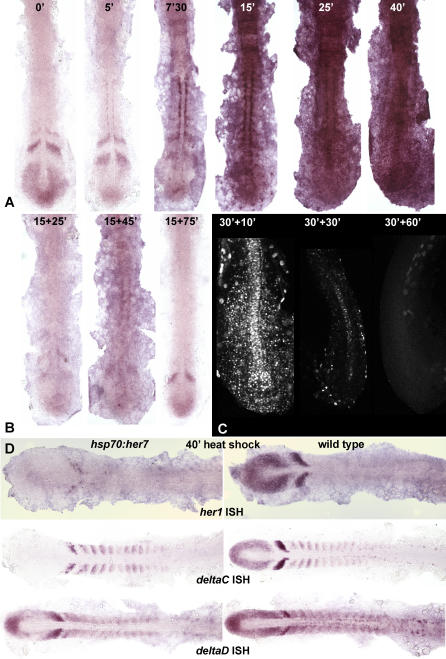
Effects of Heat Shock in *hsp70:HA-her7* Transgenics (A) Levels of *her7* mRNA expression as shown by ISH (NBT/BCIP) in embryos fixed immediately after a 37 °C heat shock for the indicated numbers of minutes. (B) Levels of *her7* mRNA in embryos given a 15-min heat shock followed by 25, 45, or 75 minutes of recovery; almost all *her7* mRNA, including the product of the normal endogenous gene, has disappeared at the 15+25-min time point, implying that Her7 inhibits *her7* expression. (C) Levels of HA-tagged Her7 protein, revealed by immunostaining with an anti-HA antibody, in embryos given a 30-min heat shock followed by 10, 30, or 60 min of recovery. (D) Effects on expression of *her1, deltaC,* and *deltaD* in embryos fixed immediately after a 40-min heat shock. *hsp70:HA-her7* transgenics are shown on the left, wild-type sibling controls on the right. All specimens in this figure have been dissected off the yolk and flat mounted.

We first checked the expression of the transgenes following heat shock, using ISH and immunochemistry to detect their transcripts and the HA-tagged protein. Figure 6 shows observations of one of the *hsp70:HA-her7* transgenic fish lines; the other lines behaved similarly. Without heat shock, the transgenic embryos showed (outside the PSM) only a barely detectable basal expression of transgenic *her7,* with expression of the endogenous *her7* gene confined to the PSM in the normal way. After 7.5 min at 37 °C, *her7* mRNA expression had clearly risen throughout the embryo; after 15 min, it was everywhere quite intense, matching the peak levels of endogenous *her7* expression normally seen in the PSM; and after 20–30 min, it reached an even higher and maximal level, such that further stay at 37 °C produced no further increase. The exogenous Her7 protein was a little slower to appear, as judged by staining with anti-HA antibody (though the difference may be partly just a consequence of the different methods of detection): it was first detected after 20 min and reached a maximal level after about 40 min of heat shock.

### The Her1 and Her7 Proteins Have Short Lifetimes

As shown in Figure 6B and 6C for an *hsp70:HA-her7* embryo heat shocked for 30 min, levels of transgene expression—both mRNA and protein—fell rapidly after the heat shock was ended and the embryo was left to recover at its normal temperature of 28 °C. The decline in mRNA levels appeared practically uniform over the whole embryo, implying that the transcripts have a similarly short lifetime in all tissues. Thus the normal pattern of variation of *her7* transcript levels in space and time is most likely achieved through regulated transcription (as our model assumes) rather than regulated degradation.

Levels of the HA-tagged protein, though still high after 10 min of recovery, were much reduced after 30 min, and undetectably low after 1 h (Figure 6C). In principle, the decay rate could be estimated from measurements of the immunolabelling fluorescence intensity at different time points; in practice, this was difficult, because the estimate depends sensitively on the amount of stain to be counted as background, and because there was substantial variability from specimen to specimen. The results for the HA-tagged protein were consistent with a lifetime as short as proposed for native Her7 and Her1 in the original model (4.3 min), though they did not exclude a value two or three times longer (unpublished data). Findings for the *hsp70:HA-her1* transgenics were similar (unpublished data).

Despite these uncertainties, it is clear that induction of the transgene is fully reversible, so that a half-hour heat shock gives rise to a brief pulse of expression of the tagged Her7 or Her1 protein, beginning within 20 min after the start of the heat shock and vanishing within an hour after the end of the shock.

### Her1 and Her7 Inhibit Transcription of Their Own Genes, of One Another, and of *deltaC*

**Figure 7:**
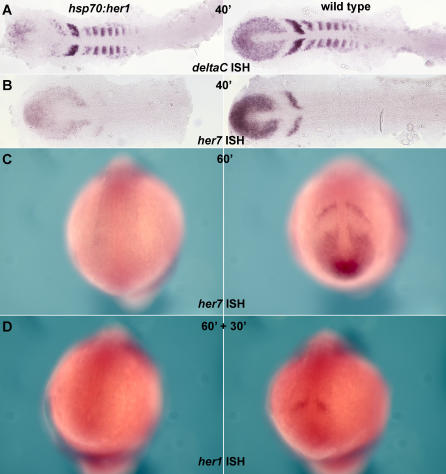
Effects of Heat Shock in *hsp70:HA-her1* Transgenics (A and B) Embryos fixed immediately after a 40-min heat shock at 37 °C, and stained by ISH for *deltaC* or *her7;* the *hsp70:HA-her1* transgenics are on the left, wild-type sibling controls on the right. The stripy expression of *deltaC* and *her7* in the PSM is reduced, but not eliminated, by the forced expression of Her1, suggesting that oscillation continues. (C) Heat shock of *hsp70:HA-her1* embryos prolonged for 60 min reduces *her7* expression to undetectable levels. (D) Heat shock of *hsp70:HA-her1* embryos for 60 min followed by 30 min recovery reveals a loss of endogenous *her1* expression, implying that *her1,* like *her7,* is autoinhibitory. (A) and (B) are flat mounts; (C) and (D) are whole mounts.

**Figure 8:**
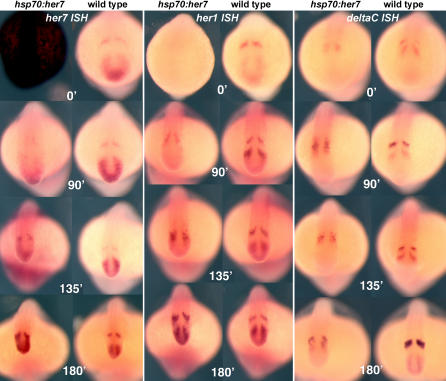
Recovery after Heat Shock in *hsp70:HA-her7* Transgenics, Compared with Wild-Type Sibling Controls All embryos were heat shocked at 37 °C for 60 min (beginning at the six-somite stage), followed by a recovery period of 0, 90, 135, or 180 min as indicated. They were then fixed and processed by ISH with probes for *her7, her1,* or *deltaC.* All these specimens are whole mounts.

We next examined how such a pulse of Her7 or Her1 overexpression would affect the expression of the endogenous oscillator genes (Figures 6, 7, and 8). Strikingly, induction of Her7 expression led within 40 min after the start of heat shock to the disappearance of all *deltaC* and *her1* transcripts from the PSM, with the notable exception of its most-anterior boundary region, where a single narrow stripe of residual expression could usually be seen (Figure 6D). In addition, *deltaC* transcripts in the formed somites were unaffected. A longer heat shock (60 min) led to disappearance of *her1* from the anterior PSM as well (see Figure 8, top row). Repression of these genes was never observed in non-transgenic heat-shocked embryos and cannot therefore be attributed to the effect of heat shock itself. These observations confirm that Her7 represses *deltaC* and *her1* transcription in the PSM. The rapidity of the effect strongly suggests that the repression is direct, and not a secondary consequence of a change in the expression of some intervening gene. We can also infer that the repressive effect of Her7 is strong only in the posterior and intermediate PSM, becomes weak in the anterior PSM, and no longer operates in formed somites. This could be because the repressive activity of Her7 depends on some partner protein whose concentration falls off with distance from the tail end of the PSM; Her13.2 or a similar protein could be a candidate for this role [37].

We could not test so directly whether Her7 represses *her7* transcription, because we did not have in situ probes that would distinguish between the closely similar transcripts of the native *her7* and the *hsp70:HA-her7* transgene. Nevertheless, using a 15-min heat shock, we observed that after 25 min of recovery at 28 °C, when heat-shock–induced transcripts from the transgene had largely disappeared, the overall level of *her7* mRNA was uniformly low, and below normal endogenous levels in the PSM (Figure 6B). This implies that the endogenous *her7* gene is down-regulated within 40 min after the onset of heat shock. Therefore, Her7 also represses its own expression in the PSM.

To test whether *her7* overexpression might lead to a general inhibition of transcription in the PSM rather than a specific inhibition of cyclically expressed genes, we also examined the expression of *deltaD,* another member of the Delta gene family expressed in the PSM and required for oscillations, but not itself oscillatory, at least in the posterior PSM. Upon induction of *her7* by a 40-min heat shock, *deltaD* transcript levels in the PSM were only slightly reduced (although with some blurring of the stripes in the anterior region); in contrast, expression of *deltaD* in proneural clusters in the neural tube was almost totally abolished (Figure 6D).

Using *hsp70:HA-her1* fish, we tested whether the induction of *her1* expression would have the same effect as for *her7*. The findings were similar, but not identical (Figure 7). Thus a 40-minute heat shock induced ubiquitous massive overexpression of Her1, and this reduced the PSM expression of *deltaC* and of *her7;* but it did not totally abolish the PSM expression of *her7*. Cyclic expression of *her7* was still evident in these fish, at apparently normal frequency, but with reduced amplitude (Figure 7A and 7B). However, a longer heat shock, of 60 min, was enough to make expression of *her7* undetectable (Figure 7C). Again, *deltaC* expression in formed somites was unaffected. Like *her7, her1* appears to be inhibited by its own protein product, because the endogenous *her1* expression pattern becomes invisible in the *hsp70:HA-her1* fish in the aftermath of the heat shock (Figure 7D).

### Transcriptional Oscillations Recover Slowly after Pulsed Overexpression of *her1* or *her7*

Several considerations suggest that *her7* is more likely than *her1* to be the key pacemaker of the transcriptional oscillations: (1) whereas morpholino knock-down of *her1* expression disrupts formation of only the first few somite boundaries, knock-down of *her7* disrupts all somite boundaries posterior to the first few [16,19,21,38]; and (2), as we have just shown, cyclic expression of *her7* seems able to continue even in the face of strong forced overexpression of *her1,* whereas all expression of *her1* seems to be lost in the face of similar forced overexpression of *her7*.

We thus predicted that a heat-shock–induced pulse of Her7 would reset the clock, and that disappearance of this exogenous Her7 would allow the expression cycle to start again in an altered phase. We therefore subjected batches of *hsp70:HA-her7* embryos to a heat shock followed by an extended recovery period at 28 °C. By about 1 h after the end of heat shock, renewed expression of *her7* in the PSM was already visible, but restoration of the normal pattern of stripes took much longer, of the order of 2 to 3 h (Figure 8).

Similar treatment of *hsp70:HA-her1* embryos again gave similar, but not identical, results. Renewed expression of *her1* mRNA was already visible 40 min after the end of heat shock, and then took more than 2 h to resolve into regular oscillations (unpublished data).

At least three factors may contribute to the slowness of recovery after heat shock. First, by transiently imposing a high level of Her7 or Her1 protein throughout the PSM, the treatment would be expected to erase the normal phase gradient responsible for the pattern of stripes; to re-establish this gradient, the anterior PSM must become populated with fresh cells that have the phase delay that results from following the normal trajectory from the posterior PSM, and this will require several hours. A second delaying factor may be the abnormal combination of regulatory molecules inside each cell at the start of the recovery period: concentrations of both mRNA and protein for *her1, her7,* and *deltaC* will all presumably be near zero, except for one member of the set of proteins—Her1 or Her7—which will be maximal. Such a condition never arises normally, and it may take some time to recover from this confused state. A third reason for the slowness of recovery lies in intercellular variation: neighbouring cells do not recover synchronously, presumably because of the variation in the heat-shock response from cell to cell and the essentially stochastic character of all gene regulation. Restoration of synchrony between desynchronised oscillators can be expected to be slow: a synchronising mechanism based on Delta-Notch signalling (or on any other short-range form of communication) will at first create microdomains within which cells are locally synchronised, but out of synchrony with the cells of the next microdomain, and this situation will then take a long time to resolve.

### Pulsed Overexpression of *her1* or *her7* Disrupts Somite Segmentation with a Five-Somite Delay

Whatever the detailed mechanics of recovery, it is clear that heat shock in the *hsp70:HA-her7* transgenics rapidly and powerfully suppresses normal expression of *her1, her7,* and *deltaC* throughout almost the entire PSM. The normal pattern of somite segmentation is thought to be controlled by the cyclic expression of these genes. We therefore expected that the heat shock would disrupt segmentation of the next somites to emerge from the PSM. But we got a surprise.

Using *hsp70:her1* and *hsp70:her7* embryos, and heat shocking them at a variety of times between the zero- and 12-somite stages, we did indeed disrupt the pattern of segmentation; but the first four or five somites to form after the beginning of the heat shock were always normally spaced. The same delay from onset of heat shock to onset of somite disruption was seen in over 100 embryos, heat shocked for times ranging from 10 min to 90 min, whatever the stage at which the heat shock was begun.

**Figure 9:**
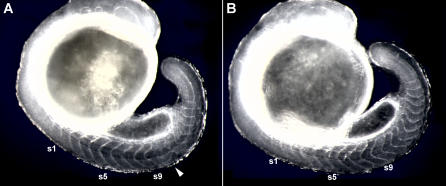
Delayed Somite Segmentation Defects following a Heat-Shock–Induced Pulse of HA-Her7 The photographs show effects of a 60-min heat shock at 37 °C initiated at the five-somite stage, followed by 7 h of recovery at the normal incubation temperature, for (A) an *hsp70:HA-her7* transgenic and (B) a wild-type sibling control. In the transgenic, four somites (s6 to s9) have formed normally after the onset of heat shock, but a block of somites subsequent to that has been disrupted (arrowhead). Embryos are lightly fixed, but unstained, and viewed with dark-field illumination.

The formation of four or five normal somites after the heat shock was followed by a disruption of segmentation in a region that varied in extent according to the duration of the heat shock and the age of the embryo when it was administered. Thus a 60-min heat shock starting at the five-somite stage (Figure 9) reproducibly caused a defect extending over five somite widths, from the level of somite 10 to that of somite 14 (*n* = 11 *hsp70:her7* embryos scored), whereas the same heat shock starting at the 12-somite stage caused a defect extending over about three somite widths, from the level of somite 17 to that of somite 19 (*n* = 8); 60-min heat shocks at the intermediate stages (*n* = 14) gave intermediate effects. Shorter heat shocks caused defects that were less extensive and not always apparent; thus, out of 64 *hsp70:her7* transgenic embryos heat shocked for 10, 20, or 30 min at the seven-somite stage, 27 showed defects, and these generally extended over only two somite widths, corresponding to the loss of one inter-somite boundary; but as always, the defects were delayed until four or five somites after the heat shock.

With all these different heat-shock regimes, somites that formed after the period of disruption appeared normal: the segmentation process eventually recovered. In sibling control embryos that we heat shocked, we saw no disturbance of segmentation, implying that the disrupted segmentation in the transgenic embryos was a specific effect of overexpression of Her1 or Her7. (Our findings here contrast with those of Roy et al. [39], who found that heat shock affected segmentation in wild-type embryos; but the segmentation defects they saw were much milder than in our transgenics, and their standard heat shock was more severe. In our hands, even a 40 °C 40-min heat shock did not cause any obvious segmentation defects in wild-type embryos, suggesting a difference between our wild-type strain and theirs.)

The five somites that form normally after a heat shock, before the segmentation defect is seen, consist of the cells that occupied the anterior half of the PSM at the time of the heat shock. (To be precise, according to our measurements at the five-somite to 12-somite stages, the distance from the posterior end of the notochord to the anterior boundary of the PSM equals 8.0 ± 1.3 [mean ± standard deviation (SD), *n* = 10] times the length of a formed somite; allowing for growth and cell movement, we calculate that the next five prospective somites occupy 46% of this region.) In the anterior PSM, evidently, the cells proceed with their segmentation programme regardless of whether *her1, her7,* and *deltaC* are oscillating or indeed expressed at all. But the occurrence of segmentation defects after the five-somite delay means that these genes do control the way the segmentation machinery of the anterior PSM is set going in the cells as they move into the anterior half of the PSM from the posterior half. And the eventual recovery of the segmentation pattern reflects the recovery of the oscillations in the posterior PSM.

## Discussion

### Detailed Measurements Support the *her1*/*7* Autoinhibition Theory of the Oscillator Mechanism

Mathematical modelling has shown that a very simple delayed negative feedback loop, based on autorepression of *her7* and/or *her1,* could account for the oscillations of gene expression that underlie somite segmentation. But the mathematics also shows that such a feedback loop can only generate oscillations if the kinetic parameters lie within appropriate ranges; if these conditions are satisfied, the period of oscillation should be primarily determined by the sum of the transcriptional and translational delays for the oscillator genes. In our original account of this model, we made rough estimates—or, in some cases, guesses—of the likely parameter values, mainly drawing on data from other gene expression systems. When we substituted these values into the model, it gave oscillations that matched the observations.

In this paper, we have shown how the beautiful spatiotemporal organization of the zebrafish somite segmentation system can be exploited so as to obtain direct measurements of several of the key parameters, including information about the delays. The measured values are close to the previous indirect estimates. We can substitute into the mathematical model (see the Supplementary Data in [25]) the new measured values of the transcriptional delays, the mRNA lifetimes (taking them to be equal to their measured upper bounds), and the translational delay for DeltaC, and re-compute the model's behaviour. To do this, we have to make an assumption about the value of *T*_init_, the time from disappearance of free inhibitory protein to the appearance of visible dots corresponding to nascent transcripts in the nucleus. If we assume *T*_init_ = 0 min, we get only damped oscillations, with a period of approximately 29 min; if we assume *T*_init_ = 3 min for each gene, we get correctly synchronized sustained oscillations with a period of 41 min, matching the period of 41 min computed using the old parameter estimates and reasonably close to the observed period of 30 min. Longer values of *T*_init_ give synchronized sustained oscillations with a longer period. Shorter values of *T*_init_ can give a similar outcome, but with a shorter oscillation period if other processes are also a little more rapid than the above estimates (but still within the margin of measurement error); for example, if we suppose that *T*_init_ = 2 min, that the mRNA lifetimes are 20% (a minute or two) shorter than our measured upper bounds, and that the DeltaC translational delay is 25 min rather than 30 min, we get synchronized sustained oscillations with a period of 36 min. Values of a few minutes for *T*_init_ are consistent with the evidence from other systems, as discussed in the previous paper [25].

There are, of course, some important parameters that remain to be measured directly, including the value of *T*_init_, the length of the translational delays for Her1 and Her7, and the protein lifetimes. Nevertheless, the measurements that we have been able to make significantly strengthen the case for believing that the mathematical model gives a true account of the oscillator mechanism of the real system. The transcriptional delays fit the proposal that autorepression of *her7* (and/or *her1*) is responsible for the observed 30-min oscillation cycle. Likewise, the observed translational delay for DeltaC, of approximately 30 min, has the magnitude required if Delta-Notch signalling is to keep the oscillations in adjacent cells synchronized in the way that the model proposes.

### Heat-Shock Experiments Establish the Logic of the Gene Control Circuitry

The proposed oscillator mechanism depends not only on kinetic parameter values, but also on the logic of the gene control circuitry. Here, too, we made assumptions, and through our heat-shock experiments in transgenic fish, we have been able to check them. Previous work [19] had shown that reduced expression of *her1* led to increased expression of *her7,* and vice versa, and that both *her* genes affected expression of *deltaC;* but the evidence that either of these *her* genes was autoinhibitory was inconclusive [38], and there was no proof that the inhibition was direct and not mediated through effects on intervening genes. Our experiments using heat shock to trigger a pulse of expression of *her1* or *her7* confirm all the postulated inhibitory actions, and the rapidity of the response to the heat shock strongly suggests that the inhibition is indeed direct in each case.

### *her1* and *her7* Regulate Segmentation Decisions in the Posterior, but Not the Anterior PSM

Lastly, our heat-shock experiments have allowed us to test the function of *her1*/*7* oscillations in controlling the pattern of somite segmentation. In particular, we have been able to discover at what time in the history of a somite the products of these genes act. Even though *her1* and *her7* normally continue to be expressed in an oscillatory fashion in the anterior half of the PSM, they exert no influence in that region on the segmentation behaviour of the cells; their influence is exerted only at an earlier stage, while the cells are in the posterior half of the PSM or in the transition zone from posterior to anterior. The delay surprised us, but it fits well with previous observations showing that the tissue in the anterior part of the PSM is already determined with respect to various other tests. Its pattern of segmentation is unaffected by treatments that interfere with FGF signalling, including treatments that cause some disruption of *her1* expression [10,12]; a block of this tissue maintains its pattern of segmentation even when rotated so as to reverse its anteroposterior axis relative to the rest of the embryo [10]; and in experiments on wild-type embryos in which heat shock was found to disrupt somite patterning, cells that lay in the anterior part of the PSM at the time of the heat shock were unaffected [39]. Our data, however, go beyond these previous observations in showing directly that the products of the oscillator genes *her1* and *her7* have no impact on the segmentation behaviour of cells in this region. Evidently, other patterning mechanisms, involving a variety of other dynamically expressed genes and cell–cell interactions [40–44], come into play in the anterior PSM. These in effect produce a delayed readout of the *her1*/*7* clock cycle phase that the cells had on leaving the posterior half of the PSM. The question of how this system of readout genes is organised and influenced by the *her1*/*7* clock is a subject for another paper (E. M. Özbudak and J Lewis, unpublished data).

### The *her1*/*7* Negative Feedback Loop Is a Strong Candidate to Be the Fundamental Pacemaker of the Somite Segmentation Clock

Several of the key assumptions of our mathematical model have yet to be tested—in particular, assumptions as to the rates of transcription and translation, and the detailed functional dependence of the rate of transcription initiation on protein concentration. Moreover, measurements of the parameters on which the model depends are not enough by themselves to prove that the *her1*/*7* oscillator is the fundamental pacemaker of the zebrafish somite segmentation clock: it remains possible, for example, that it could be only one of several loosely coupled oscillators operating in parallel [45].

In the mouse and the chick, other genes, not belonging to the Notch pathway, are indeed found to oscillate in the posterior PSM [1,3,4,9], along with homologs of the genes we have discussed here. Could it be, then, that the oscillations of *her1* and *her7* are directly driven by some other oscillator that operates independently of *her1* and *her7*? The slow pattern of recovery of *her1* and *her7* oscillation that we have described after a heat-shock–induced pulse of *her7* expression makes this unlikely. If *her1* and *her7* were merely slaves of another oscillator, their pattern of expression should recover promptly, under the command of the master oscillator, as soon as the artificial pulse of expression has disappeared; and we have seen that it does not do so, but takes about four times the normal somite cycle period to re-establish itself. The *her1*/*7* oscillator may be subject to control by another oscillator; but if so, the relationship cannot be a simple one of master and obedient slave.

The findings of this paper all lend support to our model of the mechanism of the somite segmentation clock: the logic of the control circuitry, the magnitudes of the transcriptional and translational delays, and the lifetimes of the molecules, where we have been able to measure them, all appear to be as the model supposes. If the *her7* or *her1* autorepression loop is not the fundamental pacemaker of the observed oscillations, it seems probable that it is at least capable of generating oscillations of a similar character and tempo.

The example of the somite segmentation clock shows how it is possible to analyse and explain quantitatively how the timing of at least one developmental process is controlled. The timing mechanisms of other developmental processes await investigation.

## Materials and Methods

### Heat-shock constructs and transgenesis.

The *hsp70:HA-her1* and *hsp70:HA-her7* DNA constructs consisted of 1.5 kilobases (kb) of the zebrafish *hsp70* promoter and upstream regulatory regions [46], including the short 5′ UTR, cloned upstream of *her1* (1.7 kb) or *her7* (1 kb) full-length cDNAs starting at the initial ATG, and followed by the SV40 early polyadenylation signal sequence (220 base pairs). A stretch of 33 nucleotides (GCCTACCCTTACGACGTGCCTGACTACGCTAGC) was inserted after the initiation ATG, so as to provide an influenza HA tag (AYPYDVPDYA) at the amino terminus of the produced protein. For the *hsp70:HA-her1* construct, an *EF1alpha:GFP* cassette [47] was added in reverse orientation to facilitate the identification of transgenic embryos by constitutive green fluorescent protein (GFP) expression.

The cassettes were cloned in pBluescript SK I-SceI [48] so as to be flanked by restriction sites for the homing endonuclease I-SceI. Approximately 2 nl of a solution of the resulting plasmids (15 μg/ml) were injected into freshly fertilised eggs together with I-SceI enzyme (250 μg/ml; New England Biolabs, http://www.neb.com) and 0.5% phenol red in 1× I-SceI digestion buffer (New England Biolabs).

Injected fish were raised to adulthood and screened for germline transmission, either by transgene-specific PCR on genomic DNA prepared from embryos that they spawned, or by GFP fluorescence in these progeny. Two independent lines were selected for each construct. For each line, adult transgenic fish were identified by PCR on DNA extracted from fin clips, and the transmission of the transgene was checked for Mendelian segregation (50% of the progeny of each fish inherited the transgene), thereby ensuring that integration had occurred only at a single site in the genome.

### Heat-shock procedures.

Embryos were kept at a temperature of 28 °C until the desired stage for heat shock. They were then transferred to pre-warmed E3 medium [49] in a 37 °C incubator for the desired length of time, then fixed immediately in ice-cold buffered 4% formaldehyde solution or returned to 28 °C for further development.

### In situ hybridisation and immunochemistry.

ISH was performed according to standard protocols. Digoxigenin-labelled RNA probes used were as previously described: *her1* [22], *her7* [16,19], *deltaC* [50], and *deltaD* [51]. For FISH, a peroxidase-conjugated anti-digoxigenin antibody was used (anti-DIG-POD, 1/50; Roche, http://www.roche.com), and peroxidase activity was detected using tyramide signal amplification with Alexa-488 (Molecular Probes, http://probes.invitrogen.com), FITC (PerkinElmer, http://www.perkinelmer.com), or Cy3 (Perkin Elmer) coupled to the tyramide, following manufacturer's instructions.

For dual FISH, the second RNA probe was labelled with fluorescein instead of digoxigenin, and detected using alkaline phosphatase-conjugated anti-fluorescein antibody (1/1,000; Roche) and Fast Red staining.

For co-staining of *deltaC* mRNA with DeltaC protein, we used a monoclonal antibody, zdc2, directed against the amino-terminal part of DeltaC (see below). Fixed embryos were quickly dehydrated and then rehydrated in a methanol series, then incubated with the zdc2 antibody (1/100) for 3 h, using 2 mg/ml bovine serum albumin as a blocking agent. Embryos were then incubated with a biotin-conjugated anti-mouse IgG antibody (1/200; Vector Laboratories, http://www.vectorlabs.com), before being washed and then refixed overnight in buffered 4% formaldehyde solution to stabilise the antigen–antibody complexes. Embryos were then processed for *deltaC* ISH as described above using Alexa-488–coupled tyramide to stain for anti-digoxigenin peroxidase activity, after which the peroxidase activity was destroyed by a 10-min incubation in 0.1 M glycine-HCl at pH 2.2. Biotin was then detected using biotin-streptavidin-HRP complexes (ABC kit; Vector Labs), with the HRP peroxidase activity being stained with Cy3-coupled tyramide (Perkin Elmer).

For HA tag immunodetection, we used a monoclonal rat anti-HA antibody (Roche 3F10, 1/500) in combination with Alexa-488–conjugated anti-Rat IgG secondary antibody (1/400; Molecular Probes). We used a standard immunohistochemistry protocol with short fixation in buffered 4% formaldehyde solution, permeabilisation with 1% Triton X-100, and 0.2% gelatine as a blocking agent.

All fluorescently stained specimens were counterstained with the far-red fluorescent nuclear marker TOPRO3 (Molecular Probes), flat mounted, and imaged as optical sections on a confocal microscope.

### Selection of a mouse anti-DeltaC monoclonal antibody, zdc2.

The extracellular region of zebrafish DeltaC was C-terminally fused to the rat CD4 tag and expressed as a soluble fusion protein by transient transfection of HEK293T cells. The protein was purified from tissue culture supernatant by immunoaffinity chromatography using the anti–CD4-tag monoclonal antibody OX-68 [52]. Hybridomas were generated by fusing splenocytes from immunized mice to the SP2/0 cell line. Hybridoma supernatants were screened by enzyme-linked immunosorbent assay (ELISA) to select antibodies that recognise epitopes that did not cross-react with other zebrafish Delta proteins and were also resistant to formalin fixation. Full details will be published elsewhere. The hybridoma was cloned, isotyped as a mouse IgG2a, and named zdc2.

### Image analysis.

Measurements of the geometry of the ISH patterns of *her1, her7,* and *deltaC* were made from confocal optical sections of flat-mounted embryos that were stained using tyramide signal amplification and a DNA counterstain as described above. First, images were warped using the Distort:Shear tool in Adobe Photoshop (Adobe Systems, http://www.adobe.com) to make the bands of gene expression in the anterior PSM run at right angles to the body axis. We applied the Threshold operation to the nuclear signal so that each part of the image was classified as nucleus or not nucleus. Using the Image:Calculations:Multiply tool in Photoshop, we then derived a pair of images, one showing just the ISH signal that lay within nuclei (corresponding to nascent transcripts), the other showing just the signal that did not lie within nuclei (corresponding to cytoplasmic mRNA). A graph of the mean signal intensity in the PSM for each image as a function of distance along the anteroposterior axis was obtained using the Analyze:Plot Profile tool of ImageJ. By comparing the two graphs, we determined the spatial interval from onset of the nuclear signal to onset of the cytoplasmic signal, for selected stripes in the anterior PSM (see Figure 4). We converted this to a time interval—an estimate of the transcriptional delay—using Equation 3 (see Results).

For estimation of mRNA lifetimes, we used the graph of the spatial distribution of cytoplasmic transcripts (derived as just described), smoothed the numerical data in Mathematica, subtracted the background (which we took to be the signal intensity in the minima of the graph), and from the smoothed data, measured (1/*c*)(d*c*/d*x*) in the region of steepest descent. We converted this to a lifetime as explained in Results. As a check on possible errors from nonlinearity of the staining, we performed a similar analysis on a set of ISH specimens stained with NBT/BCIP, but in this case, without discriminating between nuclear and cytoplasmic signals.

### Derivation of temporal information from the spatial wave pattern: Mathematical analysis.

Once a steady state has been reached, in which somites are formed at a steady rate through a steady production process in the PSM, we can derive a simple relationship between the observed temporal and spatial oscillations of gene expression.

Let *φ*(**x**, *t*) denote the phase of the oscillation cycle for a cell at position **x** at time *t*. If **v** is the velocity of the cell, its phase at time *t* + d*t* will be *φ*(**x** + **v**d*t*, *t* + d*t*). Thus the rate of change of phase in this cell as it moves along its trajectory (the material derivative of *φ*) is

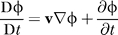



If we measure *φ* in cycles, 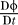 is simply the intracellular oscillation frequency in cycles per unit time; in other words,

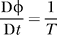
where *T* is the current value of the period of oscillation in the given cell. We assume that the rate of cycling depends only on the position of the cell relative to the tail bud (as will be the case if, for example, the rate depends only on the concentration of FGF8). We choose our origin of coordinates to be in the tail bud. Then we can write

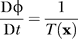



Cells reaching **x** at different times but having followed the same flowline since leaving the posterior PSM will differ in phase by an amount that simply reflects the difference in their time of exit from the posterior PSM. If cells in the posterior PSM all oscillate with period *T*_0_, it follows that

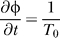
so that the spatial pattern in the PSM as a whole (posterior plus anterior) oscillates with period *T*_0_ (a snapshot of the PSM at time *t* looks the same as a snapshot at time *t* + *T*_0_, after one additional somite has emerged from the anterior end of the PSM). *T*_0_, the period of the fundamental oscillator in the posterior PSM, is thus equal to the time taken to form one extra somite, while the spatial stripes seen in the anterior PSM reflect the slowing of the oscillation in each cell as it moves out along its flowline. We can write

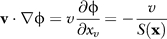
where ∂/∂*x_v_* denotes differentiation with respect to position along the flowline (parallel to **v**), and *S*(**x**) is the period of oscillation of the spatial pattern along this line. In a linear approximation, *S*(**x**) is simply the distance from peak to peak or trough to trough in the neighbourhood of **x**.

Putting all this together, we have

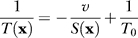



At the anterior end of the PSM, the velocity of the cells relative to the tail bud is just one somite length per somite cycle time, directed along the rostrocaudal axis, so that *v* = *S*_0_/*T*_0_, and in general, *v* = *u S*_0_/*T*_0_, where *u*(**x**) is the velocity measured in somite lengths per somite cycle time. Hence we find

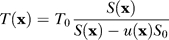
Equivalently, we can write

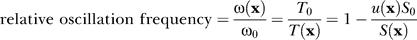
where *ω*(**x**) = 1/*T*(**x**) is the frequency of the oscillation in a cell at **x** (in cycles per unit time) and *ω*_0_ = 1/*T*_0_ .

Note that at the anterior end of the PSM, *u*(**x**) = 1 and *S*(**x**) = *S*_0_, so that *T*(**x**) → ∞, reflecting the fact that the temporal oscillation has stopped, while at the posterior end of the PSM, in the neighbourhood of the tail bud, where *u*(**x**) = 0 and *S*(**x**) is large, *T*(**x**) → *T*_0_. For the estimates shown in the main text, which are based on measurements close to the central body axis, we make a linear interpolation for the value of *u*(**x**) as a function of rostrocaudal position in the intervening region: *u*(*x*) = *x*/*L,* where *x* is the distance along the rostrocaudal axis measured from the tail end of the notochord, and *L* is the length of the PSM measured from the tail end of the notochord to the most recently formed somite boundary. The results shown in the main text are in fact not very sensitive to the exact form of *u*(*x*).

We use these formulas to read the temporal course of events from the spatial pattern as seen in the anterior PSM, where temporal cycling is still in progress (though slowing down), and the peaks and troughs of the spatial pattern are clearly defined.

## Abbreviations

FISH: fluorescent in situ hybridisation
HA: haemagglutinin
ISH: in situ hybridisation
PSM: presomitic mesoderm
s.e.m.: standard error of the mean
